# Anti-MDA5 antibody-positive dermatomyositis complicated by unilateral vocal cord paralysis: A case report

**DOI:** 10.1097/MD.0000000000051405

**Published:** 2026-10-30

**Authors:** Luan Luan, Yingying Ma, Xingjie Dong, Luying Gong, Chunfeng Hou

**Affiliations:** a Department of Rheumatology, Jining No. 1 People’s Hospital, Jining, Shandong, China; b School of Clinical Medicine, Jining Medical University, Jining, Shandong, China.

**Keywords:** anti-MDA5 positive, dermatomyositis, recurrent laryngeal nerve injury, tofacitinib, vocal cord paralysis

## Abstract

**Rationale:**

Hoarseness is a well-recognized feature in patients with anti-melanoma differentiation-associated protein 5 antibody-positive dermatomyositis (MDA5-DM); however, vocal cord paralysis (VCP) is a distinctly uncommon complication. To date, VCP has been rarely reported in MDA5-DM or other inflammatory myopathies, and the limited published cases have described exclusively bilateral cord involvement; unilateral VCP has not been documented. Although Janus kinase inhibitors have shown some effectiveness in treating refractory MDA5-DM, their therapeutic role in VCP remains unclear.

**Patient concerns:**

A 47-year-old Chinese woman with MDA5-DM had clinical improvement after treatment with glucocorticoids and cyclosporine; then she was discharged. However, 2 day after discharge, she experienced a sudden onset of hoarseness, difficulty swallowing liquids, chest tightness, and shortness of breath.

**Diagnoses:**

Flexible laryngoscopy showed immobility of the left vocal cord, consistent with left VCP. After ruling out causes such as neck surgery, trauma, and mechanical compression, we determined that the unilateral recurrent laryngeal nerve injury was related to MDA5-DM.

**Interventions:**

Because of severe gingival hyperplasia and worsening hoarseness, cyclosporine was discontinued and replaced with tacrolimus. Tofacitinib was added, along with voice therapy.

**Outcomes:**

By day 13, the patient’s hoarseness and choking had improved substantially; by day 20, these symptoms had resolved completely.

**Lessons:**

Unilateral VCP may represent a rare neurological complication in patients with MDA5-DM. For patients who present with hoarseness, difficulty swallowing liquids, or unexplained worsening of shortness of breath, laryngoscopy should be considered to facilitate early diagnosis. If calcineurin inhibitors are not tolerated or the response is inadequate, adding a Janus kinase inhibitor such as tofacitinib may be considered as part of a comprehensive immunosuppressive regimen; however, because multiple interventions were used concurrently in this case, the independent contribution of tofacitinib to neurological recovery could not be determined.

## 1. Introduction

Anti-melanoma differentiation-associated protein 5 antibody-positive dermatomyositis (MDA5-DM) is a distinct subtype of dermatomyositis characterized by skin ulcers, rapidly progressive interstitial lung disease (RP-ILD), and low muscle disease activity.^[1]^ Previous studies have shown that approximately 13.3% of patients with MDA5-DM develop hoarseness;^[2]^ however, epidemiological data on vocal cord paralysis (VCP) as the underlying cause are lacking. VCP has been rarely reported in patients with MDA5-DM or other inflammatory myopathies,^[3-5]^ and all documented cases to date have described bilateral involvement. Unilateral involvement has not been described, and its pathogenesis and clinical features remain unclear.

In recent years, tofacitinib, a Janus kinase (JAK) inhibitor that blocks the JAK-STAT signaling pathway, has demonstrated potential in treating refractory MDA5-DM and its associated RP-ILD. However, its application in MDA5-DM-related VCP has not been reported. We report a rare case of MDA5-DM complicated by unilateral VCP, review the relevant literature to explore possible mechanisms, and describe the patient’s response to tofacitinib. Our aim is to provide insights that may support early recognition and management of this uncommon complication.

## 2. Case presentation

A 47-year-old Chinese female was admitted to our hospital with a 2-week history of rash. Two weeks prior to admission, she developed a rash affecting both palms, the left upper arm, the neck, and the right buttock. This was followed by pain in both knees, both shoulders, the right toes, and the right heel. She did not report myalgia, muscle weakness, Raynaud phenomenon, or fever. Her past medical, family, and personal histories were unremarkable. Laboratory examinations revealed an antinuclear antibody titer of 1:100 and strongly positive anti-MDA5 antibody on myositis-specific autoantibody panel. The levels of immunoglobulins and complements were within the normal range. Inflammatory markers were elevated: C-reactive protein 7.57 mg/L, erythrocyte sedimentation rate 37 mm/h, and ferritin 385.1 ng/mL. Alanine aminotransferase was 79.0 U/L, aspartate aminotransferase 96.5 U/L, creatine kinase 59.1 U/L, and lactate dehydrogenase 273.0 U/L. Routine blood tests showed lymphopenia (0.94 × 10^9^/L). Hepatitis B surface antigen was positive, but hepatitis B virus-DNA was <20 IU/mL; both the T-cell spot test for tuberculosis and anti-hepatitis C virus antibody were negative. Chest computed tomography (CT) revealed interstitial lung changes (Fig. 1A–C), and pulmonary function tests were normal. A diagnosis of MDA5-DM with interstitial lung disease was made. She was treated with intravenous methylprednisolone 80 mg once daily and oral cyclosporine 50 mg twice daily, with tenofovir alafenamide 25 mg once daily added for hepatitis B virus infection. After these interventions, both the patient’s rash and her joint symptoms improved. She was released from the hospital on October 15, 2025, on a regimen involving prednisone 30 mg taken twice a day and cyclosporine. However, within 24 hours after leaving the hospital, she developed acute hoarseness, difficulty in swallowing liquids, a sensation of chest tightness, shortness of breath during exercise, and new skin eruptions on her fingers. At a tertiary hospital, her cyclosporine dose was increased to 100 mg twice a day because of the suspicion of active disease. Ten days later, her hoarseness and dysphagia had not improved and the skin rash had not cleared. She was therefore admitted back to our department on October 29, 2025.

**Figure 1.**
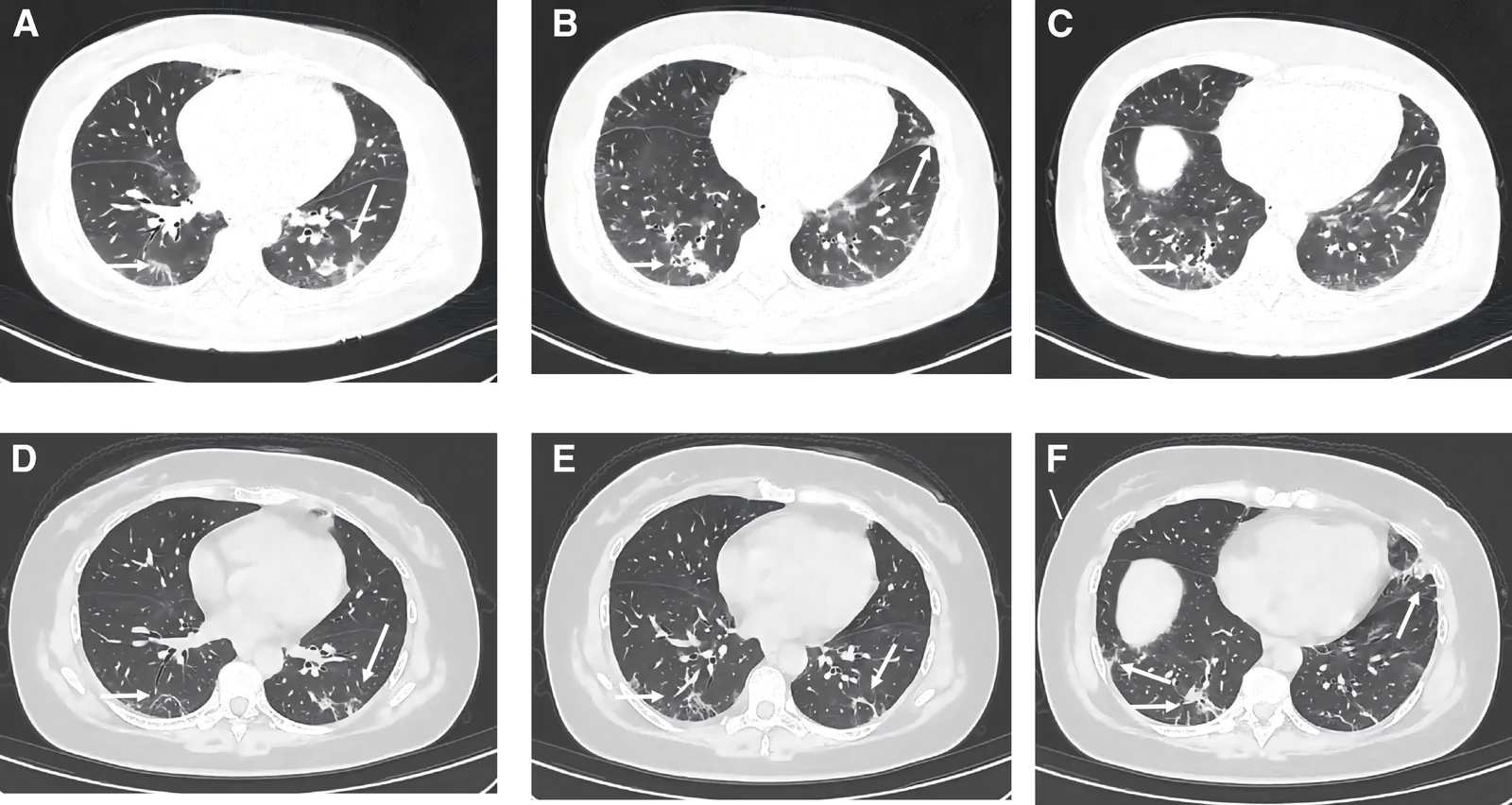
Comparison of chest CT images obtained on October 4 and October 30, 2025, at the same anatomical level, showing no significant progression of interstitial lung changes. (A–C) Chest CT (October 4, 2025). (D–F) Chest CT (October 30, 2025). CT = computed tomography

### 2.1. Physical examination

Hoarseness was noted. Erythematous rashes were present on the medial right eyelid, the face, the palmar aspects of both hands, and the volar surfaces of the fingers, accompanied by a few vesicles, and on the extensor surfaces of both elbows, the left upper arm, and the right buttock, with crusting in some areas (Fig. 2).

**Figure 2.**
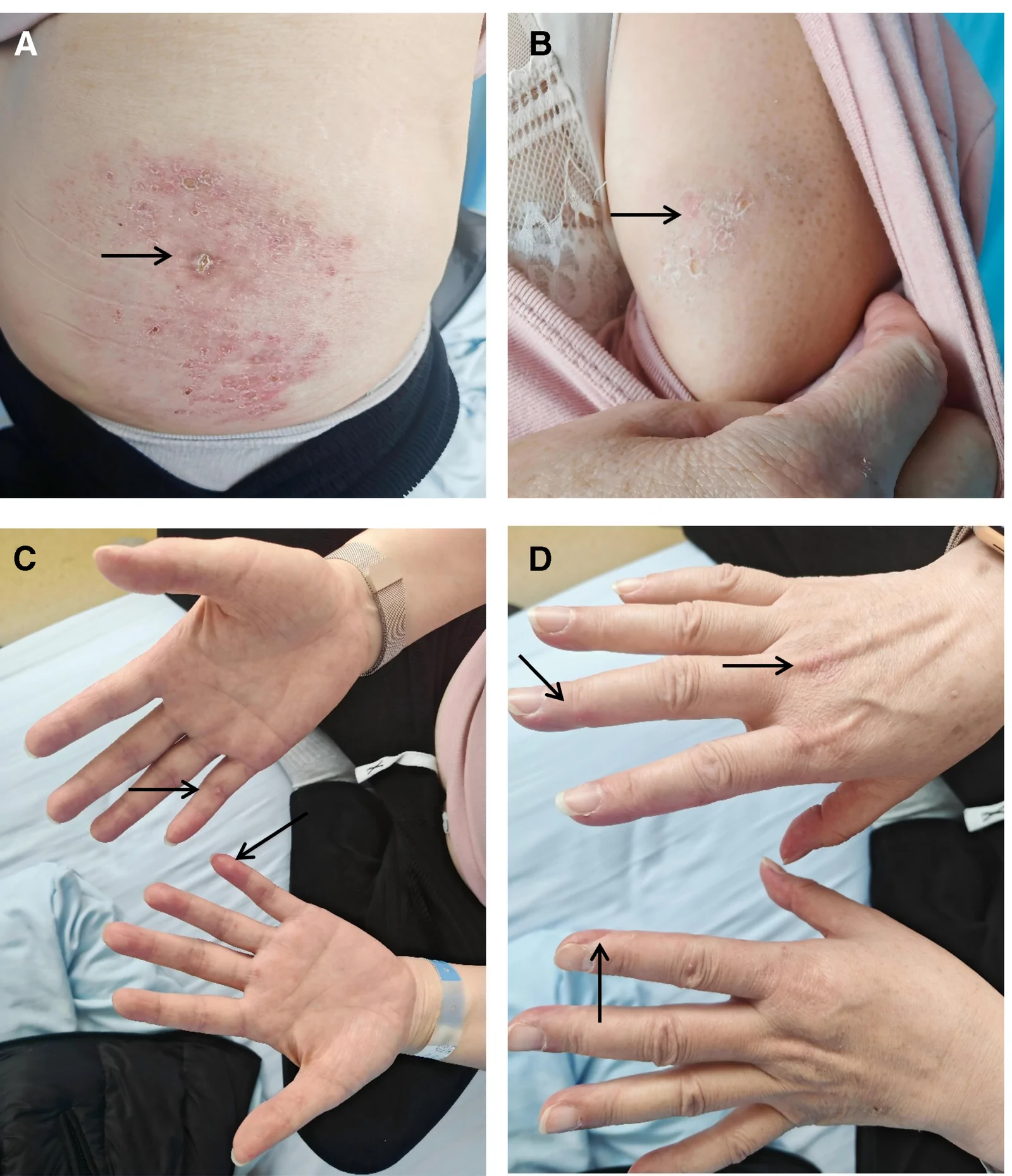
Cutaneous manifestations observed in the patient. (A) Erythematous rash with ulceration on the right buttock; (B), Erythematous rash on the left elbow; and (C, D) Rashes on the fingers.

### 2.2. Investigations

Arterial blood gas analysis, routine blood tests, erythrocyte sedimentation rate, and electrolytes were unremarkable. Liver enzymes were elevated: alanine aminotransferase was 109.6 U/L, aspartate aminotransferase was 52.4 U/L, and gamma-glutamyl transferase was 94.5 U/L. Creatine kinase was 27.9 U/L. The cyclosporine trough level was 142.2 ng/mL. Flexible laryngoscopy showed a normal-appearing epiglottis, smooth mucosa over the epiglottis, bilateral piriform sinuses, and the postcricoid region, without obvious mass lesions. The bilateral vocal fold mucosa was smooth, with no masses. The left vocal fold was fixed in abduction, whereas the right vocal fold moved normally. A glottic gap was observed during closure, consistent with left VCP (Fig. 3). Non-contrast CT of the neck revealed no space-occupying lesions. Ultrasonography of the thyroid and cervical lymph nodes, echocardiography, and electrocardiogram were all normal. Repeat chest CT showed no progression compared with the study performed on October 4 (Fig. 1D–F).

**Figure 3.**
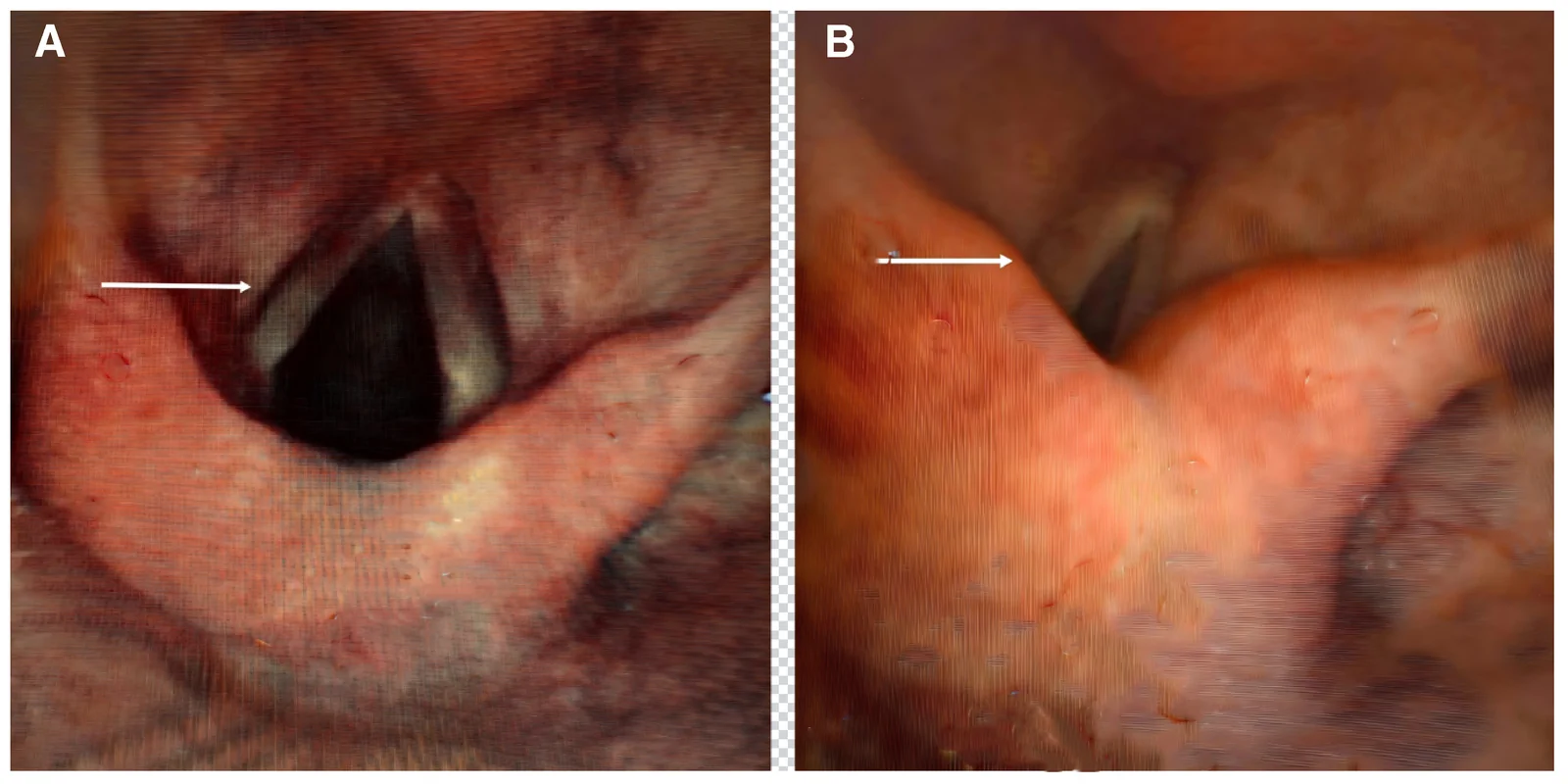
Flexible laryngoscopy shows the left vocal fold fixed in the abducted position, whereas the right vocal fold moves normally, and a glottic gap is observed during closure. (A) Inspiratory phase and (B) phonatory phase.

Even after increasing the cyclosporine dose, the patient’s symptoms did not improve, and she developed severe gingival hyperplasia that was difficult to tolerate. As a result, cyclosporine was discontinued and replaced with tacrolimus 1 mg twice daily. The repeat chest CT scan showed no progression of interstitial lung disease, and both electrocardiography and echocardiography were normal. After excluding acute exacerbation of interstitial lung disease and cardiac causes, and based on the laryngoscopic findings, we attributed her chest tightness and hoarseness to VCP. Since there was no history of neck surgery, trauma, or mass compression, we diagnosed the patient with MDA5-DM-related VCP. Because the new-onset VCP occurred despite high-dose glucocorticoid and calcineurin inhibitor (CNI) therapy, we added tofacitinib 5 mg twice daily to the maintenance regimen of intravenous methylprednisolone 60 mg once daily. We also added speech rehabilitation training, conducted simultaneously. After 13 days of this treatment, her chest tightness, shortness of breath, and difficulty swallowing liquids improved significantly, and her hoarseness decreased. She was discharged in a stable and improved condition.

We conducted a telephone follow-up 7 days after discharge; the patient was satisfied with the treatment results and reported that her hoarseness and difficulty swallowing liquids had completely resolved. Since her symptoms had fully improved, she declined a further laryngoscopic examination to assess recovery of vocal cord movement. At the time of this report, she remained clinically stable and had not experienced any adverse events related to tofacitinib.

## 3. Discussion

Hoarseness is a common symptom in otolaryngology practice, with etiologies including acute and chronic laryngitis, functional dysphonia, neoplasms, and neurogenic causes.^[6]^ Although laryngeal involvement in systemic diseases is relatively uncommon, it should be suspected in the context of autoimmune disorders. For instance, VCP secondary to rheumatoid arthritis or amyloidosis has been reported.^[6]^ Recent research suggests a significantly higher prevalence of voice symptoms in patients with autoimmune diseases: 16.6% of these patients have a Voice Handicap Index-10 score > 7, a rate significantly higher than that in healthy controls, suggesting that autoimmune disease is an independent risk factor for voice symptoms.^[7]^ Cricoarytenoid joint involvement, vasculitis affecting the recurrent laryngeal or vagus nerve, mechanical compression by pulmonary hypertension, diffuse laryngeal edema, and rheumatoid nodules involving the vocal cords may be the possible mechanisms.^[8-11]^ The characteristics of laryngeal involvement in MDA5-DM remain to be clarified. Existing data indicate that the incidence of hoarseness in patients with MDA5-DM is approximately 13.3%.^[2]^ A prospective study by Huang et al further revealed that 26.7% of patients with MDA5-DM had laryngopharyngeal involvement, among whom 62.9% presented with hoarseness and 31.4% had concomitant dysphagia.^[12]^ Laryngoscopy was performed in all patients with laryngopharyngeal involvement and showed glottic closure insufficiency in 34.3%, but the underlying cause was not further evaluated.^[12]^ Currently, data are lacking on the exact etiology and prevalence of VCP in MDA5-DM. No systematic study of VCP in MDA5-DM or other inflammatory myopathies exists; only isolated case reports are available. The reported cases have had bilateral involvement, with laryngoscopic findings of bilateral vocal cord fixation in abduction accompanied by extensive laryngeal muscle atrophy, or diffuse bilateral vocal cord hyperemia and edema with white plaques extending from the vocal process to the anterior commissure and absent mucosal vibration.^[3-5]^ These patients may present with hoarseness, dysphonia, dyspnea, and dysphagia on clinical examination. In contrast, our patient presented with unilateral (left) VCP without white plaques on the vocal fold surface, a pattern that differs markedly from the previously described bilateral involvement. Filaire et al reported that 87% of patients with surgically transected recurrent laryngeal nerves had the vocal cord fixed in abduction.^[13]^ Together with the laryngoscopic findings, we speculate that the lesion may be limited to the unilateral recurrent laryngeal nerve pathway. Unfortunately, laryngeal electromyography (LEMG) was not available at our institution to further differentiate between neurogenic and myogenic injury patterns. An important limitation of this report is the lack of LEMG or other neurophysiological studies to objectively confirm recurrent laryngeal nerve neuropathy. Therefore, the findings in this case remain presumptive and should be interpreted with caution.

The mechanism of VCP in MDA5-DM has not been fully elucidated. Possible explanations include the following. First, direct involvement of laryngeal muscles by the disease may lead to submucosal muscle necrosis; this typically presents as bilateral VCP,^[3-5]^ which is inconsistent with the unilateral involvement observed in our patient. Second, neural injury (either central or peripheral) may be responsible. Central lesions can directly damage the high vagus nerve or compress and stretch the vagus nerve trunk and its branches, resulting in unilateral or bilateral VCP.^[14]^ The higher the level of vagus nerve injury, the more severe the symptoms tend to be. High-level vagus nerve lesions are often accompanied by multiple cranial nerve involvement and extensive laryngopharyngeal dysfunction and gastrointestinal symptoms.^[15-17]^ Peripheral lesions, in contrast, primarily affect the recurrent laryngeal nerve and present as isolated VCP. Our patient presented predominantly with hoarseness and choking on liquids without other neurological deficits. Together with the laryngoscopic finding of the left vocal cord fixed in abduction, this is more consistent with peripheral injury to the left recurrent laryngeal nerve.^[13]^ Cranial magnetic resonance imaging was therefore not performed. Among the common causes of unilateral recurrent laryngeal nerve palsy, tumor compression is the most frequent (32%), followed by surgical injury (30%).^[18]^ Other potential causes of unilateral VCP include occult malignancy, viral neuritis, diabetic neuropathy, and medication-related neurotoxicity. The patient had no history of neck surgery. Two consecutive chest CT scans revealed no mediastinal or apical pulmonary masses, and echocardiography excluded pulmonary hypertension, thereby ruling out mechanical compression and surgical injury. The patient exhibited no fever or prodromal symptoms indicative of viral infection, had no history of diabetes, and demonstrated normal blood glucose levels. She was not receiving any neurotoxic medications. Consequently, viral neuritis, diabetic neuropathy, and drug-induced neuropathy were considered unlikely. Although idiopathic recurrent laryngeal nerve palsy and other inflammatory neuropathies unrelated to dermatomyositis could not be entirely excluded, the presence of anti-MDA5 antibodies and the clinical context of active dermatomyositis suggested that immune-mediated recurrent laryngeal nerve injury associated with MDA5-DM was the most probable etiology.

The pathogenic role of anti-MDA5 antibodies in VCP remains incompletely understood, but several mechanisms may be postulated. First, anti-MDA5 antibody titers correlate closely with disease activity and severity. The antibody activates the type I interferon (IFN) pathway, promotes neutrophil extracellular trap release, and amplifies IFN-stimulated gene expression, creating a pro-inflammatory microenvironment that may damage the vasa nervorum of the recurrent laryngeal nerve. Second, the recurrent laryngeal nerve – particularly the left side, which courses around the aortic arch – is anatomically vulnerable to inflammatory or vasculitic processes in the mediastinum and neck. Focal perineural inflammation or small-vessel vasculitis mediated by anti-MDA5 immune complexes could therefore preferentially affect this nerve. Third, compared with bilateral VCP, unilateral VCP suggests a more localized injury rather than diffuse brainstem or generalized neuromuscular involvement, consistent with a mononeuropathy occasionally reported in dermatomyositis. However, without histopathological confirmation from the affected nerve tissue, this proposed mechanism remains speculative.^[19]^

Possible mechanisms of immune-mediated nerve injury include the following: First, overactivation of the type I IFN pathway.^[1]^ In an autopsy study of patients with COVID-19, Suh et al^[20]^ found that 25.7% of the deceased had peripheral nerve inflammation, and 77.8% of nerve biopsy specimens showed endothelial expression of the type I IFN activation marker myxovirus resistance protein A, suggesting that an excessive type I IFN response may independently trigger immune-mediated nerve injury. Although COVID-19 and MDA5-DM differ in etiology, both share dysregulation of the type I IFN pathway as a core pathological feature. It is therefore plausible that overactivation of this pathway leads to immune-mediated nerve injury through cytokine dysregulation. In this case, the patient developed VCP while receiving high-dose glucocorticoid and CNI therapy, which suggests that conventional treatment might not sufficiently block the type I IFN pathway. The rapid reversal of symptoms after adding a JAK inhibitor provides indirect clinical evidence consistent with this mechanism. Unfortunately, due to financial constraints, the patient declined repeat measurement of anti-MDA5 antibody titers. Serial antibody measurements, particularly if titers had shown a downward trend following tofacitinib initiation, would have provided stronger evidence supporting a direct pathogenic contribution of anti-MDA5 antibodies to VCP. Second, vasculitic neuropathy. The hallmark pathological feature of dermatomyositis is complement-mediated small-vessel vasculitis, and deposition of the C5b-9 membrane attack complex can lead to microangiopathy.^[21]^ Dhawan et al^[22]^ confirmed by nerve biopsy that dermatomyositis can be associated with large-and small-vessel vasculitis. Gwathmey et al^[23]^ identified dermatomyositis as a definite cause of neuropathy secondary to systemic vasculitis. It should be noted that the 2 pathogenetic mechanisms may coexist or be mutually exclusive; type I IFN-driven vasculitis may act alongside direct immune attack to cause inflammation of the vasa nervorum and impair neural conduction. Previous reports have indicated that glucocorticoids and intravenous immunoglobulin alone have unsatisfactory efficacy for VCP associated with inflammatory myopathies.^[5]^ In our patient, the addition of tofacitinib resulted in marked improvement of hoarseness and choking on liquids by day 13, with complete resolution of symptoms by day 20 (7 days after discharge). Functional recovery from VCP can occur through 2 mechanisms: functional compensation by the contralateral vocal cord, or recovery of neural conduction following resolution of inflammation. However, structural compensation or recovery of vocal cord function usually requires at least 6 months.^[24,25]^ Our patient achieved complete resolution within 20 days, which is far shorter than the time required for structural compensation. This result suggests that the symptom improvement is primarily due to recovery of neural conduction following control of inflammation, rather than to structural compensation of the vocal cords. Tacrolimus and cyclosporine are both CNIs with similar mechanisms of action, and the main reason for switching from cyclosporine to tacrolimus was intolerance to gingival hyperplasia. As a JAK inhibitor, tofacitinib suppresses type I and type II IFN signaling as well as interleukin-21 signaling,^[19]^ thereby precisely targeting the core immunopathology of anti-MDA5-DM – overactivation of the type I IFN pathway. Nevertheless, the patient also received glucocorticoids, tacrolimus, and voice rehabilitation. Because multiple therapeutic interventions were initiated simultaneously, we cannot determine the independent contribution of tofacitinib to neurological recovery. A synergistic effect of multiple interventions remains possible. Future controlled studies with larger sample sizes are needed to clarify the independent effect of each intervention. A recent case report from Soğur et al details a fatal instance of MDA5-DM which was complicated by RP-ILD and reactivation of cytomegalovirus. This report once again shows the wide range of severe and atypical manifestations linked to this autoantibody and suggests that anti-MDA5 positivity should be regarded as a high-risk biomarker, calling for early risk stratification, early administration of immunomodulatory therapy, and careful monitoring for opportunistic infections.^[26]^

## 4. Conclusion

The case of unilateral VCP confirmed by laryngoscopy in a patient with MDA5-DM indicates that unilateral VCP may be a rare neurological complication of this condition. Insufficient detection and evaluation may be the most important reason for the apparent “rarity” of VCP in dermatomyositis. Laryngoscopy is generally performed in dermatomyositis patients only when overt otorhinolaryngological manifestations develop. Subtle voice changes or dysphagia are frequently attributed to esophageal dysmotility, glucocorticoid-induced myopathy, or interstitial lung disease, which masks laryngeal pathology. This case shows that for patients who exhibit even mild dysphonia or swallowing difficulties, proactive laryngoscopic evaluation is important, as early detection may alter management and prevent complications. Unilateral VCP often has an insidious clinical presentation and is easily overlooked, unlike bilateral VCP, which may rapidly progress to life-threatening manifestations. In the present case, the patient had already developed hoarseness and choking on liquids, which impaired her quality of life. Unilateral VCP can also lead to aspiration, impaired cough reflex, and further compromise of airway protective mechanisms. In patients with MDA5-DM, who are predisposed to RP-ILD, this may represent an important factor that accelerates disease progression. For patients with MDA5-DM who develop hoarseness, difficulty swallowing liquids, or unexplained worsening of shortness of breath, routine laryngoscopy should be performed to enable early detection of VCP and to prevent misdiagnosis as progression of interstitial lung disease. When available, LEMG should also be performed to confirm neurogenic VCP and differentiate it from myogenic injury. In this case, a comprehensive treatment approach that includes tofacitinib may be appropriate. Further studies evaluating each therapeutic modality independently are needed to clarify tofacitinib’s specific role in promoting neurological recovery. However, this study is limited by its single-center design, the lack of LEMG data, and the lack of long-term follow-up. Further research with larger patient cohorts and comprehensive neurophysiological evaluation is needed to elucidate the underlying mechanisms and to ascertain optimal treatment strategies for MDA5-DM-related VCP.

## Acknowledgments

We thank the patient for permission to publish this case report.

## Author contributions

**Conceptualization:** Luan Luan, Chunfeng Hou.

**Investigation:** Luan Luan, Xingjie Dong, Luying Gong.

**Supervision:** Yingying Ma.

**Writing – original draft:** Luan Luan.

**Writing – review & editing:** Yingying Ma, Chunfeng Hou.

## References

[1] Lu X Peng Q Wang G. Anti-MDA5 antibody-positive dermatomyositis: pathogenesis and clinical progress. Nat Rev Rheumatol. 2024;20:48–62. 10.1038/s41584-023-01054-9 38057474

[2] So H So J Lam TT. Performance of the 2017 European alliance of associations for rheumatology/american college of rheumatology classification criteria in patients with idiopathic inflammatory myopathy and anti-melanoma differentiation-associated protein 5 positivity. Arthritis Rheumatol. 2022;74:1588–92. 10.1002/art.42150 35467787

[3] Wood MW McNeill E Mims JW Wright SC Jr. Dysphonia and dermatomyositis: an unusual manifestation. Laryngoscope. 2013;123:460–2. 10.1002/lary.23540 23042610

[4] Althagafi HO Morishita K Petty RE. Severe vocal cord dysfunction: an unusual complication of juvenile dermatomyositis. J Rheumatol. 2013;40:744–5. 10.3899/jrheum.121338 23637383

[5] Alsaed OS Alamlih LI Farfar KA Mohd TRN Alemadi SAAR Malallah HA. Vocal cord palsy in refractory anti-melanoma differentiation-associated protein 5 antibody-positive dermatomyositis. Rheumatology (Oxford). 2020;59:e66–7. 10.1093/rheumatology/keaa130 32252065

[6] Reiter R Hoffmann TK Pickhard A Brosch S. Hoarseness-causes and treatments. Dtsch Arztebl Int. 2015;112:329–37. 10.3238/arztebl.2015.0329 PMC4458789 26043420

[7] Liu ZW Masterson LM Srouji IA Musonda P Scott DGI. Voice symptoms in patients with autoimmune disease: a cross-sectional epidemiological study. Otolaryngol Head Neck Surg. 2012;147:1108–13. 10.1177/0194599812459024 22931896

[8] Kadura S Raghu G. Rheumatoid arthritis-interstitial lung disease: manifestations and current concepts in pathogenesis and management. Eur Respir Rev. 2021;30:210011. 10.1183/16000617.0011-2021 PMC9489133 34168062

[9] McNellis EL Hoang KG. Immunosuppression and systemic lupus erythematosus predisposing to laryngeal abscess. Otolaryngol Head Neck Surg. 1997;116:107–9. 10.1016/S0194-59989770359-0 9018267

[10] Lechner M Sutton L Fishman JM. Otorhinolaryngology and diving-part 2: otorhinolaryngological fitness for compressed gas scuba diving: a review. JAMA Otolaryngol Head Neck Surg. 2018;144:259–63. 10.1001/jamaoto.2017.2616 29450499

[11] Aszkenasy OM Clarke TJ Hickling P Marshall AJ. Systemic lupus erythematosus, pulmonary hypertension, and left recurrent laryngeal nerve palsy. Ann Rheum Dis. 1987;46:246–7. 10.1136/ard.46.3.246 PMC1002109 3579390

[12] Huang W Ren F Deng D. The clinical characteristics of pharyngeal and laryngeal lesions in anti-MDA5-positive dermatomyositis patients. Clin Exp Rheumatol. 2025;43:276–81. 10.55563/clinexprheumatol/t0478a 39625817

[13] Filaire M Mom T Laurent S. Vocal cord dysfunction after left lung resection for cancer. Eur J Cardiothorac Surg. 2001;20:705–11. 10.1016/s1010-7940(01)00819-3 11574212

[14] Subspecialty Group of Laryngopharyngology; Editorial Board of Chinese Journal of Otorhinolaryngology Head and Neck Surgery; Subspecialty Group of Laryngopharyngology. [Experts consensus on diagnosis and treatment of vocal fold paralysis]. Zhonghua Er Bi Yan Hou Tou Jing Wai Ke Za Zhi. 2021;56:198–209. 10.3760/cma.j.cn115330-20200924-00765 33730801

[15] Best SR Ahn J Langmead S. Voice and swallowing dysfunction in neurofibromatosis 2. Otolaryngol Head Neck Surg. 2018;158:505–10. 10.1177/0194599817741839 29160153

[16] Kang K Moon BG. Developmental abnormalities of the craniocervical junction resulting in Collet-Sicard syndrome. Spine J. 2016;16:e635–9. 10.1016/j.spinee.2016.04.022 27157500

[17] Malagelada C Keller J Sifrim D. European guideline on chronic nausea and vomiting-a UEG and ESNM consensus for clinical management. . United Eur Gastroenterol J. 2025;13:427–71. 10.1002/ueg2.12711 PMC11999049 39754724

[18] Ramadan HH Wax MK Avery S. Outcome and changing cause of unilateral vocal cord paralysis. Otolaryngol Head Neck Surg. 1998;118:199–202. 10.1016/S0194-5998(98)80014-4 9482553

[19] Yoshida T Nakashima R. Anti- melanoma differentiation-associated gene 5 antibody positive dermatomyositis: recent progress in pathophysiology and treatment. Curr Rheumatol Rep. 2025;27:23. 10.1007/s11926-025-01188-7 PMC12052924 40323493

[20] Suh J Mukerji SS Collens SI. Skeletal muscle and peripheral nerve histopathology in COVID-19. Neurology. 2021;97:e849–58. 10.1212/WNL.0000000000012344 34099523

[21] Clark KEN Isenberg DA. A review of inflammatory idiopathic myopathy focusing on polymyositis. Eur J Neurol. 2018;25:13–23. 10.1111/ene.13357 28816394

[22] Dhawan PS Dyck PJB Tracy JA Naddaf E. Systemic vasculitis with dermatomyositis, hearing loss, neuropathy, and multiorgan dysfunction. Neurology. 2017;89:2119–20. 10.1212/WNL.0000000000004656 29133608

[23] Gwathmey KG Burns TM Collins MP Dyck PJB. Vasculitic neuropathies. Lancet Neurol. 2014;13:67–82. 10.1016/S1474-4422(13)70236-9 24331794

[24] Husain S Sadoughi B Mor N Sulica L. Time course of recovery of iatrogenic vocal fold paralysis. Laryngoscope. 2019;129:1159–63. 10.1002/lary.27572 30325515

[25] Husain S Sadoughi B Mor N Levin AM Sulica L. Time course of recovery of idiopathic vocal fold paralysis. Laryngoscope. 2018;128:148–52. 10.1002/lary.26762 28736846

[26] Soğur OA Gokten DB Mercan R. Anti-MDA5 dermatomyositis with RP-ILD and CMV reactivation: a case report. Rom J Intern Med. 2026;64:149–54. 10.2478/rjim-2026-0004 41914513

